# The Roles of TIF1γ in Cancer

**DOI:** 10.3389/fonc.2019.00979

**Published:** 2019-10-02

**Authors:** Chengpeng Yu, Zeyang Ding, Huifang Liang, Bixiang Zhang, Xiaoping Chen

**Affiliations:** Hepatic Surgery Center, Tongji Hospital, Tongji Medical College, Huazhong University of Science and Technology, Wuhan, China

**Keywords:** TIF1γ, cancer, TGFβ/Smad signaling, Wnt/β-catenin signaling, DHX33-NLRP3 signaling

## Abstract

Transcriptional intermediary factor 1 γ (TIF1γ), also known as TRIM33, RFG7, PTC7, or Ectodermin, is an E3 ubiquitin-ligase family member with a ring-box-coiled-coil region. It can regulate TGF-β/Smad signaling in two different ways in different cellular contexts. On one hand, TIF1γ can monoubiquitinate Smad4 to inhibit the formation of Smad2/3/4 nuclear complexes. On the other hand, TIF1γ can function as a cofactor of phosphorylated (p)-Smad2/3, competing with Smad4 to inhibit the formation of the Smad2/3/4 complex. In addition, TIF1γ has been reported to play a role in transcription elongation, cellular differentiation, embryonic development, and mitosis. As transforming growth factor-β (TGF-β) superfamily signaling plays an important role in the occurrence and development of cancer, and TIF1γ was reported to be involved in the regulation of TGF-β superfamily signaling, studies on TIF1γ during the last decade have focused on its role in the development of cancer. However, TIF1γ can function either as a tumor suppressor or promoter in different cellular contexts, yet there are few reviews focusing on the roles of TIF1γ in cancer. Hence, in this paper we systematically review and discuss the roles of TIF1γ in cancer. Firstly, we review the biological features, the regulatory mechanisms and the related signaling pathways of TIF1γ. Next, we illustrate the roles of TIF1γ in different tumors. We then provide a tentative hypothesis that explains the dual roles of TIF1 γ in cancer. Finally, we provide our viewpoint regarding the future developments of cancer research focusing on TIF1γ, especially in relation to the effects of TIF1γ on tumoral immunity.

## Introduction

Transcriptional intermediary factor 1 γ (TIF1γ), synonymous with TRIM33, RFG7, PTC7, or Ectodermin, is an E3 ubiquitin-ligase family member with a ring-box-coiled-coil region (1). It has been reported to play a role in transcription elongation (2, 3), DNA repair (4), differentiation of cells (2, 5, 6), embryonic development (6–9), mitosis (10), and dermatomyositis (11–13). As transforming growth factor-β (TGF-β) superfamily signaling plays an important role in the occurrence and development of cancer (14–16), and TIF1γ was reported to be involved in the regulation of TGF-β superfamily signaling (17, 18), recent studies on TIF1γ have focused on its role in tumorigenesis (10, 19–24).

Perplexingly, TIF1γ can function either as a tumor suppressor or promoter in different cells. In many different tumors, such as non-small-cell lung cancer, breast cancer, glioma, and clear cell renal cell carcinoma (21, 23, 25, 26), TIF1γ acts as a tumor suppressor and its expression is decreased. However, in B lymphoblastic leukemia, pancreatic cancer, and cervical carcinoma (10, 27, 28), TIF1γ functions as a tumor promoter and prevents the apoptosis of tumor cells. However, there are few reviews focusing on the dual and contradictory roles of TIF1γ in cancer. We therefore systematically review and discuss the roles of TIF1γ in cancer in this paper. Firstly, we review the biological features, the regulatory mechanisms and the related signaling pathways of TIF1γ. Next, we illustrate the roles of TIF1γ in different tumors. We then provide a tentative hypothesis that explains the dual roles of TIF1 γ in cancer. Finally, we provide our viewpoint regarding the future developments of cancer research focusing on TIF1γ, especially in relation to the effects of TIF1γ on tumor immunity.

## The Biological Functions of TIF1γ

**Figure 1:**
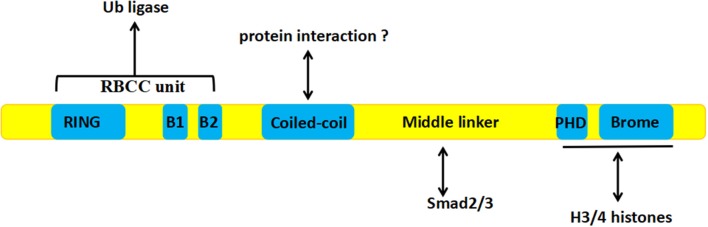
The structure of TIF1γ: The RBCC unit at the N terminus is involved in ubiquitination, A PHD domain and a bromodomain at the C terminus can interact with histones 3 and 4, and the middle linker can interact with activated Smad2 and Smad3.

TIF1γ is a 123 kDa protein consisting of 1120 amino acids encoded by the *trim33* gene, which is 118,415 bps in length and contains 21 exons and 20 introns, encoded on chromosome 1 in humans (29). The TIF1γ protein consist of several different domains. At the N terminus, there is a ring-box-coiled-coil (RBCC) unit, containing a RING domain, B boxes, and a coiled-coil domain, which is involved in the ubiquitination of Smad4 (30), TGF-beta1 receptor (TβRI) (31), and β-catenin (23), as well as the sumoylation of SnoN1 (32). A PHD domain and a bromodomain at the C terminus can interact with histones 3 and 4 (33, 34). Between these regions, there is a middle linker which can interact with activated Smad2 and Smad3. The middle linker is less well-conserved, which explains why the other members of the TIF1γ family cannot interact with Smad proteins [Figure 1; (33)].

## The Regulatory Mechanisms of TIF1γ

**Figure 2:**
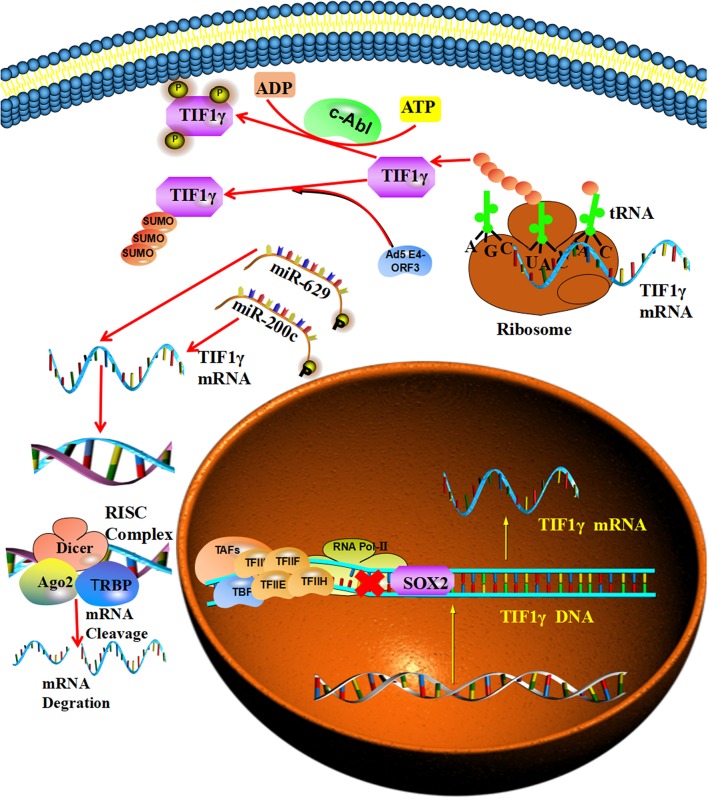
The regulatory mechanisms of TIF1γ: SOX2 can bind to the putative SRY-binding sites of the TIF1γ promoter and repress its expression. MiR-200c and miR-629 can bind to specific sequences in the 3′-UTR of TIF1γ mRNA and promote its degradation. The c-Abl tyrosine kinase can regulate the tyrosine phosphorylation of TIF1γ. In addition, the Ad5 E4-ORF3 protein can promote the initial SUMO3 conjugation to TIF1γ and induce its sumoylation.

SRY-related HMG-box2 (SOX2) was reported to be involved in the transcriptional regulation of TIF1γ and can bind to the putative SRY-binding sites of the TIF1γ promoter, which represses the expression of TIF1γ at the mRNA and thus protein level (21). Furthermore, Jingushi et al. reported that miR-629 is involved in the post-transcriptional regulation of TIF1γ and can bind to a specific sequence in the 3′-UTR of TIF1γ mRNA and promote the degradation of TIF1γ mRNA (25). At the same time, miR-429/miR-200b-3p was also reported to be involved in the post-transcriptional regulation of TIF1γ and to be able to bind to a specific sequence in the 3′-UTR of TIF1γ mRNA, which promotes its degradation. Additionally, the circular RNA PTK2 can bind directly to miR-429/miR-200b-3p to protect TIF1γ mRNA from targeting by miR-429/miR-200b-3p (35). Moreover, Yuki et al. reported that v-abl Abelson murine leukemia viral oncogene homolog 1(c-Abl) tyrosine kinase takes part in the post-translational regulation of TIF1γ and can regulate its phosphorylation at tyrosines 524, 610, and 1,048, which inhibits the interaction of TIF1γ with Smad2/3 (36). At the same time, the Ad5 E4-ORF3 protein can promote the initial conjugation of SUMO3 to TIF1γ, inducing its sumoylation and proteasomal degradation [Figure 2; (37, 38)].

## The Physiological Roles of TIF1γ

As a transcriptional intermediary factor, TIF1γ takes part in the transcriptional regulation of a number of genes by interacting with other transcriptional factors. For example, TIF1γ can promote the transcription elongation of hematopoietic genes by interacting with FACT, p-TEFb, and the SCL complex, and TIF1γ deficiency reduced the full-length transcript level of these genes (2). At the same time, TIF1γ controls hematopoiesis and the specification of the germ layer and regulates cell growth by antagonizing TGFβ signaling (2, 7). Furthermore, TIF1γ can promote the repair of DNA damage by interacting with Amplified in Liver Cancer 1 (ALC1) in a poly(ADP-ribose) polymerase (PARP)-dependent manner (4).

## Signalings Pathways Related to TIF1γ in Cancer

### The Inhibitory Effect of TIF1γ on TGF-β/Smad Signaling

**Figure 3:**
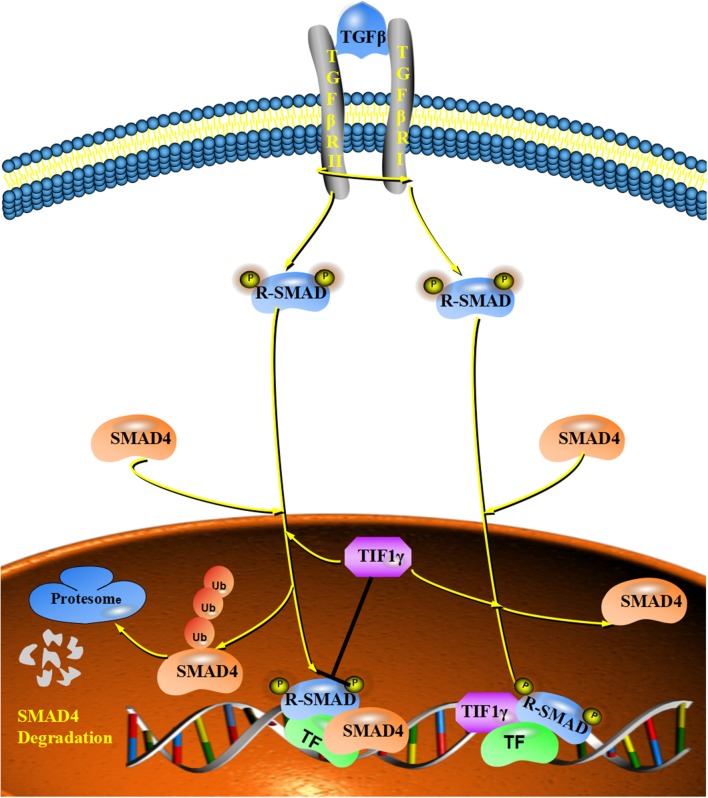
The inhibitory effect of TIF1γ on TGF-β/Smad signaling: TIF1γ can regulate TGF-β/Smad signaling in two different ways. On the one hand, it can monoubiquitinate Smad4 and inhibit the formation of Smad nuclear complexes. On the other hand, TIF1γ can function as a cofactor of phosphorylated (p)-Smad2/3, competing with Smad4 to inhibit the formation of the Smad2/3/4 complex.

TGF-β plays a vital role in the regulation of cellular proliferation, differentiation, apoptosis, motility, invasion, and immune responses (18, 39–41). Smad proteins can be phosphorylated by TGF-β and translocate to the nucleus, which results in the transcriptional activation of downstream target genes (32, 42, 43). Increasing numbers of studies show that TGF-β/Smad signaling is involved in tumor growth, metastasis and the epithelial–mesenchymal transition (EMT) (44–47). Specifically, TGFβ/Smad signaling can function as a tumor suppressor to inhibit tumor growth and metastasis by regulating the downstream genes, such as p21, p53, c-myc, and snail (48, 49). Deletions or mutations of TGFβ/Smad signaling were detected in many cancers (48). For example, mutations of Smad2 were found in cervical cancer, colorectal cancer and hepatocellular carcinoma (50–52). At the same time, mutations of Smad4 are more frequent in some cancers, such as colon cancer, gastric cancer, and pancreatic tumors (53–55). TIF1γ can regulate TGF-β/Smad signaling in two different ways in different cellular contexts. On the one hand, it can monoubiquitinate Smad4 and inhibit the formation of Smad nuclear complexes (7, 30). On the other hand, TIF1γ can function as a cofactor of phosphorylated (p)-Smad2/3, competing with Smad4 to inhibit the formation of the Smad2/3/4 complex (17). Additionally, TIF1γ requires sumoylation mediated by ubiquitin carrier 9 to exert its inhibitory effect on TGFβ/Smad4 signaling (24, 37). Numerous studies have demonstrated that TIF1γ can inhibit tumor growth, TGF-β-induced epithelial mesenchymal transition and metastasis, and that its expression is reduced in non-small-cell lung cancer and breast cancer [Figure 3; (21, 22, 35)]. However, FAM/USP9x can reverse the ubiquitination of Smad4 and counteract the activity of TIF1γ in TGF-β/Smad signaling (30). Moreover, forkhead box M1 (FOXM1) can also counteract the activity of TIF1γ in TGF-β/Smad signaling by interfering with the interaction between TIF1γ and Smad4 (26). In addition, αB-crystallin can interact with TIF1γ and disrupt the monoubiquitination of Smad4, which favors the formation of the Smad2/3/4 complex and enhances TGF-β/Smad signaling (56).

### The Inhibitory Effect of TIF1γ on Wnt/β-Catenin Signaling

**Figure 4:**
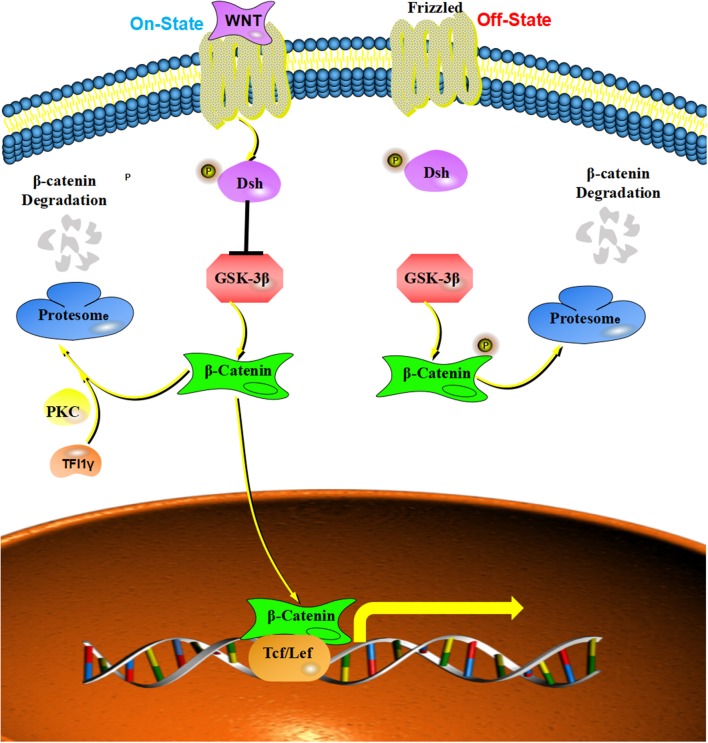
The inhibitory effect of TIF1γ on Wnt/β-catenin signaling: Wnt protein can protected β-catenin from being phosphorylated by disrupting the “destruction complex,” and TIF1γ can ubiquitylate nuclear β-catenin with the assistance of protein kinase Cδ (PKC) to inhibit Wnt/β-catenin signaling.

The Wnt signaling pathway exerts an important role in regulating stem cell self-renewal, cell proliferation, differentiation, adhesion, and migration (57–61). Wnt protein can protect β-catenin from being phosphorylated by disrupting the “destruction complex” of β-catenin (62–64), which can enable β-catenin to translocate to the nucleus and form a complex with TCF/LEF (T-cell specific transcription factor/lymphoid enhancer-binding factor), which induces the expression of Wnt-targeted genes (65–67). Wnt/β-catenin signaling can influence tumor growth and metastasis by regulating the expression of the downstream genes, such as c-myc, cyclin D1, and Snail (68). Increasing numbers of studies report the dysregulation of Wnt/β-catenin signaling in many human cancers (68). For example, increased expression of Wnt ligands was detected in colon cancer, breast cancer and lung cancer (53, 69, 70). Furthermore, mutations of β-catenin were founded in colon cancer, gastric cancer, and hepatocellular carcinoma (69, 71, 72). Moreover, TIF1γ was reported to regulate Wnt/β-catenin signaling by interacting with and ubiquitylating nuclear β-catenin with the assistance of protein kinase Cδ, which degrades nuclear β-catenin and inhibits cell proliferation and tumorigenesis in glioblastoma (23). These studies provide new insights into the development of human cancers caused by aberrant activation of β-catenin (Figure 4).

### The Positive Effect of TIF1γ on DHX33-NLRP3 Signaling

**Figure 5:**
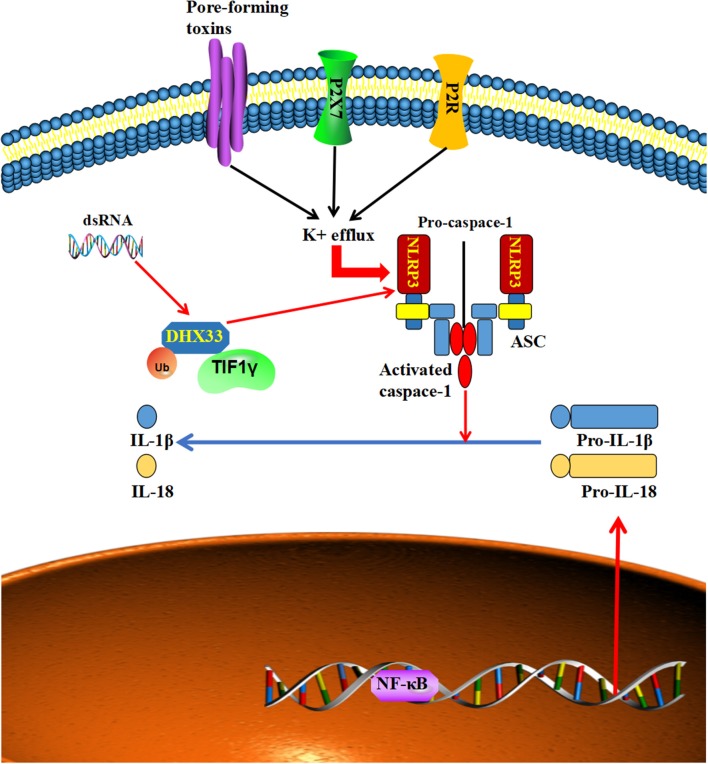
Multiple types of stimulatory signals can activate NLRP3 to form a macromolecular signaling complex with its adaptor protein ASC, which causes the cleavage of pro-caspase-1 to the active form of caspase-1, which in turn transforms pro-IL-1β and pro-interleukin (IL)-18 into their biologically active, mature secreted forms. Furthermore, TIF1γ can ubiquitinate and assist DHX33 to interact with, and activate NLRP3 to form a macromolecular signaling complex to produce mature IL-1β and IL-18.

Nod-like receptor 3 (NLRP3) is a member of the eponymous receptor family, which can perceive multiple types of stimulatory molecules (73–76), such as ATP, crystalline reagents and the microbial toxin nigericin, and form a macromolecular signaling complex with its adaptor protein ASC and procaspase-1 to induce inflammasome assembly (77–80). DHX33, a member of the DExD/H-box helicase family, is a cytosolic RNA sensor that can bind to and activate NLRP3 to oligomerize and recruit the adaptor protein ASC and cause the cleavage of pro-caspase-1 to the active form of caspase-1 (81, 82). Caspase-1 then transforms pro-IL-1β and pro-interleukin (IL)-18 into their biologically active mature secreted forms to induce inflammation (83, 84). There is increasing evidence that the expression of the NLRP3 inflammasome is dysregulated in many cancers, such as head and neck squamous cell carcinoma, hepatocellular carcinoma, and colorectal cancer (71, 85, 86). Furthermore, the overactivation of NLRP3 was related to poor survival and tumor invasiveness in head and neck squamous cell carcinoma and breast cancer (85, 87). At the same time, NLRP3 inflammasome takes part in the resistance to radiotherapy and chemotherapy in oral squamous cell carcinoma and glioblastoma (88, 89). While the activation of the NLRP3 inflammasome complex needs the assistance of TIF1γ, TIF1γ can bind to and ubiquitinate DHX33 at lysine 218, which helps DHX33 activate NLRP3 under dsRNA stimulation (90). Accordingly, a knockdown of TIF1γ disrupted the dsRNA-induced NLRP3 inflammasome activation in macrophages (90). Furthermore, increasing numbers of studies demonstrate that the NLRP3 inflammasome plays a vital role in the metastasis of tumors (89, 91, 92). Taken together, these results imply that TIF1γ might influence the metastasis of tumors via DHX33-NLRP3 signaling (Figure 5).

## The Roles of TIF1γ in Different Types of Tumors (Table 1)

### The Expression of TIF1γ in Tumors

The expression of TIF1γ varies in different tumors. The expression of TIF1γ is low in most tumors, such as liver cancer, pancreatic cancer, lung cancer, renal carcinoma, and glioblastoma (20, 21, 23, 25, 27). However, its expression is increased in some tumors, such as colorectal cancer and breast cancer (Table 1). At the same time, the roles of TIF1γ also vary in different tumors. In some tumors, TIF1γ functions as a tumor promoter and prevents the apoptosis of tumor cells, but it also acts as a tumor suppressor in other tumors and inhibits the growth of tumor cells.

**Table 1:** The dysregulated expression and target genes of TIF1γ in cancer.

**Type of cancer**	**Subtype of cancer**	**TIF1γ expression**	**Reasons for dysregulation in cancer**	**Involved signaling pathways**	**References**
Liver cancer	Hepatocellular carcinoma	Down	Hypermethylation	TGF-β signaling	(20)
Pancreatic cancer	Pancreatic ductal adenocarcinoma	Down	–	TGF-β signaling	(19)
Lung cancer	Non-small cell lung cancer	Down	SOX2 miR-429/miR-200b-3p	TGF-β signaling	(21, 35)
Leukemia	Chronic myelomonocytic leukemia	Down	Hypermethylation	TGF-β signaling	(93)
Renal carcinoma	Renal cell carcinoma	Down	miR-629	TGF-β signaling	(25)
Glioblastoma	Glioblastoma multiforme	Down	–	Wnt-β-catenin	(23)
Colorectal cancer	–	Up	–	TGFβ/Smad4	(79)
Breast cancer	–	UpDown	–	TGF-β signaling	(26) (22)
Cervical carcinoma	–	–	c-Abl	TGF-β signaling APC/C	(10, 36)

*TGF-β, transforming growth factor-β; SOX2, SRY-related HMG-box2; c-Abl, v-abl Abelson murine leukemia viral oncogene homolog 1; APC/C, anaphase-promoting complex/cyclosome*.

### TIF1γ in Liver Cancer

In one of our own earlier studies (20), we found that the CpG islands in the TIF1γ promoter were hypermethylated and the expression of TIF1γ was reduced in hepatocellular carcinoma (HCC), especially in samples from advanced HCC. At the same time, the decreased expression of TIF1γ was an independent and significant risk factor for recurrence and survival after curative resection (20). Furthermore, the combined measurement of TIF1γ and p-Smad2 was found to be a more powerful predictor of poor prognosis in HCC patients. Interestingly, TIF1γ plays a double role in HCC cells. It favors tumor growth in early-, but not in advanced-stage HCC. However, TIF1γ inhibits the invasion and metastasis of both early- and advanced-stage HCC. Mechanistically, TIF1γ can suppress TGF-β/Smad signaling by monoubiquitinating Smad4 and inhibiting the formation of the Smad2/3/4 complex to regulate tumor growth and metastasis. Specifically, TIF1γ can relieve TGFβ-induced growth inhibition and favor tumor growth in early-stage HCC. In advanced-stage HCC, TIF1γ in turn inhibits TGF-β-induced tumor invasion and metastasis. Furthermore, we confirmed that the downstream cascades of TGF-β/Smad signaling, such as c-myc, p21/cip1, p15/ink4b, and protein kinase B–signaling transactivation, are also downregulated by TIF1γ (20). At the same time, another study reported that TIF1γ can interact with TIF1α and TIF1β to form a regulatory complex that suppresses murine hepatocellular carcinoma (94). Furthermore, TIF1γ can also interact with TIF1α to inhibit VL30 retrotransposons, and thus plays an important role in retroviral restriction and antiviral defense, which broadens what is known about the roles of the TRIM family of proteins in the endogenous retrovirus (ERV)-derived oncogenic regulatory network (95).

### TIF1γ in Pancreatic Cancer

TIF1γ expression was reported to be decreased in pancreatic cancer tissues (19, 27, 96). At the same time, the expression of TIF1γ was inversely correlated with Smad 4 expression in pancreatic cancer cell lines and the overexpression of TIF1γ suppressed TGFβ signaling to inhibit the growth and invasion of pancreatic cancer cells (27). Furthermore, TIF1γ inactivation was found to cooperate with Kras^G12D^ activation to induce cystic pancreatic tumors that resemble human intraductal papillary mucinous neoplasms (19).

### TIF1γ in Colorectal Cancer

TIF1γ was found to be overexpressed in colorectal cancer and its expression levels were found to be associated with advanced tumor stage (7, 79). Furthermore, the expression of TIF1γ attenuated TGF-β-induced growth inhibition (7). At the same time, increased TIF1γ expression was correlated with a loss of Smad4 in colorectal cancer and predicted a poor prognosis for colorectal cancer patients (79). However, another study reported that the knockdown of TIF1γ resulted in genomic instability and cancer progression in colorectal cancer by regulating mitotic checkpoints (28). At the same time, TIF1γ was reported to interact with ALC1 (Amplified in Liver Cancer 1) and is involved in DNA repair in a Poly (ADP-ribose) polymerase 1 (PARP1)-dependent manner (4). Furthermore, Shi et al. reported that the loss of TIF1γ in colorectal cancer cell lines can cause resistance to the bromodomain and extraterminal domain (BET) protein inhibitors via MYC and TGF-β-dependent mechanisms (97). This further implies that TIF1γ also acts as a tumor suppressor in colorectal cancer. However, the underlying mechanisms that can explain the contradictory results require further research.

### TIF1γ in Breast Cancer

TIF1γ expression was reported to be slightly reduced in human breast cancer tissues, compared to normal breast tissues. Moreover, the overexpression of FOXM1 in breast cancer can interact with Smad3/Smad4 and inhibit the binding of TIF1γ to Smad4 to prevent its ubiquitination, which can attenuate the inhibitory effects of TIF1γ on TGF-β signaling to promote the metastasis of breast cancer (26). However, another study reported that TIF1γ expression was increased in 35.9 % of breast cancer patients and its expression was related to younger age, estrogen receptor (ER) negativity, and tumors larger than 2 cm. Additionally, TIF1γ overexpression was related to poor prognosis in breast cancer patients (22), but the contradictory results require more thorough investigation. Furthermore, the deletion of TIF1γ was found to enhance TGFβ-induced growth inhibition in breast cell lines via Smad4 in MDA-MB468 signaling (7). In addition, TIF1γ can inhibit the EMT of mammary epithelial cells and terminal differentiation of mammary alveolar epithelial cells by antagonizing Smad4 (9, 47).

### TIF1γ in Lung Cancer

TIF1γ was reported to be decreased in non-small cell lung cancer (NSCLC), but the CpG islands in the TIF1γ promoter were not found to be hypermethylated (98). Furthermore, Wang et al. reported that the expression of TIF1γ was downregulated by the overexpression of SOX2 in NSCLC tissues (21), and the reduced expression of TIF1γ was associated with poor survival of the patients (35). Furthermore, knockdown of TIF1γ was found to promote TGF-β-induced EMT and invasion of NSCLC cells *in vitro* and favor their metastasis. Conversely, the knockdown of SOX2 attenuated TGF-β-induced EMT and invasion of NSCLC cells. At the same time, expression of the circular RNA PTK2 was reported to be decreased in metastatic NSCLC tissues compared to non-metastatic NSCLC tissues, and was found to protect TIF1γ from miR-429/miR-200b-3p-mediated downregulation (35). Finally, the overexpression of circPTK2 was found to promote TIF1γ expression and suppress TGF-β-induced EMT and NSCLC cell invasion (35).

### TIF1γ in Chronic Myelomonocytic Leukemia

TIF1γ was reported to be downregulated in a subset of chronic myelomonocytic leukemia (CMLL) patients (93, 99), and tif1g^Δ/Δ^ mice were confirmed to develop a CMML-like myeloproliferative disease with monocytic features. Furthermore, TIF1γ was found to regulate the differentiation of hematopoietic progenitor populations (17) and promote the expansion of the granulomonocytic progenitor compartment. At the same time, the response of hematopoietic cells to TGF-β is suppressed in tif1γ^Δ/Δ^ mice (93). Finally, the CpG sequences of TIF1γ were found to be hypermethylated and a demethylating agent recovered the normal epigenetic status of the TIF1γ promoter and the expression of TIF1γ in human cells (93), which implies that TIF1γ is an epigenetically regulated tumor suppressor gene in hematopoietic cells.

### TIF1γ in Other Tumors

TIF1γ expression was found to be decreased and associated with pathological stages and grades in clear cell renal cell carcinoma, and the overexpression of TIF1γ inhibited the growth and invasion of its tumor cells (25). At the same time, TIF1γ expression was decreased and inversely correlated with the levels of β-catenin Ser715 phosphorylation in primary glioblastoma multiforme (GBM) specimens, and the overexpression of TIF1γ inhibited the growth of GBM cells by destabilizing β-catenin (23). However, TIF1γ can promote tumor cell survival by being recruited by PU.1 to bind to two lineage-specific enhancers near the Bim gene and antagonizing PU.1 function in B lymphoblastic leukemia (B-ALL) cells (100). At the same time, TIF1γ can also favor the proliferation of tumor cells by binding to the anaphase-promoting complex/cyclosome (APC/C) to promote the mitosis in HeLa cells (10). In addition, the knockdown of TIF1γ can enhance the TGF-β-induced elongation of HeLa cells (36).

## A Tentative Hypothesis That Explains the Dual Roles of TIF1γ in cancer

As presented in this review, TIF1γ can function either as a tumor suppressor or promoter according to the different cellular contexts. For example, TIF1γ acts as a tumor suppressor and inhibits the tumor growth of non-small-cell lung cancer (35). By contrast, TIF1γ act as a tumor promoter in B lymphoblastic leukemia and can prevent the apoptosis of tumor cells (100). The potential underlying mechanism that causes these contrary results remains a mystery. We hypothesized that the different functions of TIF1γ might be attributed to inconsistent experimental results. On the one hand, as an intermediary transcriptional factor, TIF1γ can regulate the transcription of target genes. For example, TIF1γ was found to be recruited by PU.1 to bind to two lineage-specific enhancers near the *Bim* gene to antagonize PU.1 function and promote the survival B-ALL cells (100, 101). On the other hand, as an E3 ubiquitin-ligase family member, TIF1γ can monoubiquitinate targeted proteins leading to their degradation. For example, TIF1γ can monoubiquitinate Smad4 and suppress TGFβ signaling to inhibit the growth and invasion of pancreatic cancer cells (27). However, the mechanisms underlying the dual roles of TIF1γ in other tumors still require further research.

## Prospects and Conclusions

As TIF1γ was reported to regulate the fate and differentiation of hematopoietic cells, increasing studies have reported on the roles of TIF1γ in immunity (17, 102–104). Ferri et al. reported that TIF1γ can be recruited by PU.1 to bind to the *Ifnb1* Control Element (ICE) and regulate the chromatin structure of the interferon-β gene (*Ifnb1*), which suppresses its transcription by preventing the recruitment of CBP/p300 in the late phase of macrophage activation (105). Additionally, TIF1γ was also reported to regulate the production and activation of macrophages (106). At the same time, it was also reported that TIF1γ is involved in and regulates macrophage motility (107). TIF1γ was also reported to regulate the differentiation of granulomonopoiesis in mice (108). Furthermore, it was reported that TIF1γ also controls the lineage expansion of invariant natural killer T (iNKT) cells (109). In addition, TIF1γ is involved in the differentiation and development of T helper 17 (Th17) cells and can decrease the production of IL-10 to regulate the cells' proinflammatory function (110), and numerous studies have demonstrated that Th17 cells, macrophages, and iNKT cells play important roles in antitumor immunity (111–117). Taken together, it can be speculated that TIF1γ might be involved in tumoral immunity and this question certainly merits further investigation in future studies.

The abnormal expression of TIF1γ has been evidenced in many kinds of tumors and plays a vital role in cancer progression and metastasis (19, 21, 25). Furthermore, TIF1γ might become a potential prognostic marker for cancer patients. For example, increased TIF1γ expression predicted a poor prognosis for colorectal cancer patients (20, 79). At the same time, TIF1γ might become a potential therapeutic target for cancer treatment. Abundant evidence demonstrates that TIF1γ is downregulated and plays an important tumor suppressive role in multiple types of cancer (19, 20, 23). Our team also showed that lentivirus-mediated TIF1γ overexpression could inhibit the invasion and metastasis of HCC cells *in vivo* (20). However, the exact mechanisms underlying the dual roles of TIF1γ in cancer are still unclear. Moreover, the functions of other domains of TIF1γ, such as the B boxes and the coiled coil domain, also remains unclear. Solving these problems will help us better understand the conflicting roles of TIF1γ in cancer.

## Author Contributions

XC and BZ provided direction and guidance throughout the preparation of this manuscript. CY, ZD, and HL wrote and edited the manuscript. All authors read and approved the final manuscript.

### Conflict of Interest

The authors declare that the research was conducted in the absence of any commercial or financial relationships that could be construed as a potential conflict of interest.
